# A Community-Based Assessment of Hypertension and Some Other Cardiovascular Disease Risk Factors in Ngaoundéré, Cameroon

**DOI:** 10.1155/2016/4754636

**Published:** 2016-12-14

**Authors:** Olivier Pancha Mbouemboue, Diana Derew, Jacques Olivier Ngoufack Tsougmo, Marcel Tangyi Tamanji

**Affiliations:** ^1^Department of Biomedical Sciences, Faculty of Science, University of Ngaoundéré, P.O. Box 454, Ngaoundéré, Cameroon; ^2^General Medicine Service, Ngaoundéré Regional Hospital, P.O. Box 45, Ngaoundéré, Cameroon; ^3^Clinical Laboratory Service, Ngaoundéré Regional Hospital, P.O. Box 45, Ngaoundéré, Cameroon; ^4^Faculty of Science, University of Buea, P.O. Box 63, Buea, Cameroon

## Abstract

*Background and Objective*. Cardiovascular diseases are primary causes of death worldwide with well documented risk factors whose varying impacts added to the complexity in CVD management dictate the need for region-specific studies. We aimed at investigating the interactions between CVD risk factors and hypertension in Ngaoundéré.* Methods*. A cross-sectional survey was carried out from March to August 2014. Sociodemographic, fasting blood glucose, blood pressure, and anthropometric data were recorded. Statistical analyses were carried out using SAS software version 9.1.* Results*. 700 adults resident in Ngaoundéré for at least two years consented and were included in the survey. Abdominal obesity, physical inactivity, and hypertension were the dominant risk factors recording 51.1%, 35.4%, and 20.4%, respectively. The prevalence of hyperglycaemia, tobacco consumption, obesity, and alcohol consumption was 5.6%, 8.3%, 9.6%, and 18.1%, respectively. Advanced age, hyperglycaemia, a divorced marital status, and alcohol consumption were independent determinants of high blood pressure.* Conclusion*. Physical inactivity, abdominal obesity, and hypertension were the most prevalent CVD risk factors, and the role of advanced age and hyperglycaemia in the occurrence of high blood pressure was reiterated. Health programs need to focus on effective screening, prevention, and control of CVDs in the Adamawa Region and Cameroon at large.

## 1. Introduction

Noncommunicable diseases (NCDs) represent an essential public health problem worldwide and their increasing occurrence in Africa simultaneously with infectious diseases is of particular importance [1, 2]. Generally, NCDs account for approximately two-thirds of the global mortality with diabetes and cardiovascular pathologies among other diseases taking the lead [3, 4]. NCDs and cardiovascular diseases (CVDs) contribute, respectively, to 82% and 37% of all the deaths under the age of 70 in developing countries [4]. CVDs represent the first cause of mortality and are responsible for about a third of all deaths worldwide, four-fifths of which occur in developing countries [4, 5]. In Cameroon, CVDs account for 11% of total mortality and central to this problem are associated risk factors defined by WHO as any attribute, characteristic, or exposure of an individual that increases the likelihood of developing the disease [6]. Consequently, CVDs prevention and the reduction of associated mortality will not be effective without direct interventions on these factors including population based investigations on the distribution of and interactions between risk factors, sensitisation, and strengthening awareness of the general population on CVDs prevention needs [7–10]. Although a majority of CVD risk factors are well known and established, the variability of the individual contribution of each risk factor in different communities and ethnic groups, as well as the complexity of the management of CVD patients, necessitates the conduction of specific regional and subregional surveys, worldwide.

Data on the distribution of cardiovascular risk factors in the Adamawa Region of Cameroon is insufficient, forming the basis for this work in investigating and collecting current data on cardiovascular disease risk factors in Ngaoundéré, the principal town of the region, with the aim of contributing to the reduction of cardiovascular risk and prevention of CVDs.

## 2. Study Area

**Figure 1:**
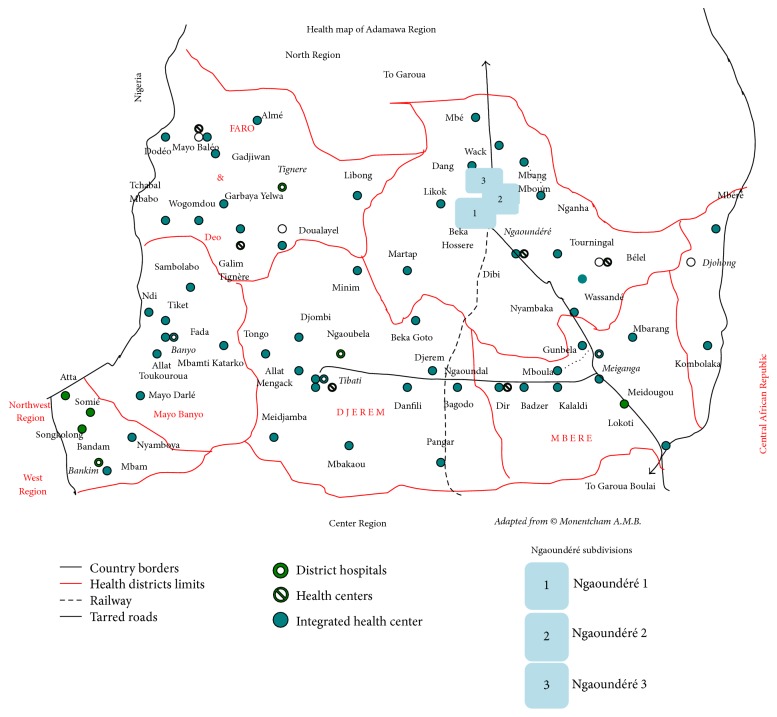
Map of the Adamawa Region of Cameroon, Africa.

Our survey was carried out in Ngaoundéré, the headquarter of the Adamawa Region (Figure 1). This region shares boundaries in the south and north with the centre and north regions of Cameroon, respectively, in the west with Nigeria, and in the east with the Central African Republic. It is found in the high altitude guinea savannah ecological zone of Cameroon, possessing a surface area of 62000 km^2^, and is characterised by two seasons: a rainy season spanning the period from April to October and a dry season from November to March [11]. The main economic activity in the region is dairy farming and agriculture.

Ngaoundéré town comprises three subdivisions. The main health structure is the Ngaoundéré Regional Hospital which is public, followed by a private protestant hospital. There are also one subdivisional medical centre, one social medical centre at the Ngaoundéré University, four integrated health centres, and many private health centres. The population is made up of several ethnic groups of unequal proportions and distribution among which are the Foulbes, the Bororo, the Haoussas, the Gbaya, the Tikar, the Mboum, and the Dii.

## 3. Methods

### 3.1. Study Design, Subjects, and Selection Criteria

We conducted a prospective cross-sectional community-based survey from March to August 2014. We enrolled persons at least 18 years of age who resided in the study area for at least two years and who provided written consent to participate in the survey. Pregnant women and seriously sick persons (bedridden or in terminal phase of chronic disease) were not included in our study.

### 3.2. Sample Size

The sample size was calculated according to the following Lorenz formula:

(1) $$\begin{array}{c} N=\frac{t^{2}\times p\left(1-p\right)}{m^{2}}, \end{array}$$

where *N* is the required sample size, *t* is *Z*-score, in our case 1.96 for 95% confidence level, *p* is the estimated prevalence of the risk factor in the survey zone, and *m* is the error margin, in our case 0.05 for 5%.

### 3.3. Sampling Method

We performed a two-stage sampling procedure wherein the quarters within the three subdivisions of the town were initially stratified following socioeconomic characteristics (low, middle, and high class and university area, considering majority of inhabitants). At least one quarter was selected from each subdivision to approximately represent the overall socioeconomic quotas; thus, 7 quarters have been selected. Secondly, households were selected within each quarter using a systematic method where every *K*th household was selected beginning with that of the quarter head until the allocated number of participants per quarter attended. The calculated value of *K* varied depending on the average number of households and inhabitants per household in each quarter. Seven quarters were selected and sensitised with the help of quarter head messengers and community relay workers prior to data collection, after which 100 participants were recruited per quarter.

### 3.4. Sample and Data Collection Procedure

In the course of our study, all adults per households visited who fulfilled eligibility criteria of the study were enrolled without any discrimination upon their gender or religious, ethnic, or social status. All data were collected and measurements performed in the evenings from 4 p.m. to 6 p.m. at the participant's residence except for fasting blood glucose (FBG) which was measured between 6 a.m. and 9 a.m. the following morning for the same participants from whom data were collected the day before. Participants who could not communicate in French were assisted with translations to the Fulfulde (local) dialect which is widely spoken in the region. We prepared and pretested a semistructured questionnaire with which information on sociodemographics and several epidemiological factors and determinants of cardiovascular disease was collected.

### 3.5. Measured Variables

#### 3.5.1. Blood Pressure (BP)

Arterial blood pressure was measured with the help of a manual mercury sphygmomanometer (ADC Prosphyg model 770) according to the STEPS method described by the WHO [12]. This was performed following a 15-minute rest period, with the individual assuming a sitting position and on both arms, and having an average time lapse of 3 to 5 minutes between paired measurements. Arterial blood pressure measurements of 140/90 mmHg or higher were considered as high blood pressure and results were recorded. Participants with high blood pressure measurements were referred for consultation with a cardiologist for comprehensive examination including other risk factors out of the scope of our study.

#### 3.5.2. Fasting Blood Glucose (FBG)

Fasting blood glucose levels were equally measured following WHO recommendations after an 8–12-hour overnight fast, using a glucometer (Accu-Chek® Performa). Hyperglycaemia was defined according to the WHO threshold [12], with measurements being greater than 1.26 g/dL.

#### 3.5.3. Weight

We measured participants' weights using a mechanical balance (mark: SECA), with light weight clothing and ensuring that the scale was on a flat and stable surface. The measurement of the body weight was done in conformity with the WHO STEPS method [12].

#### 3.5.4. Height

Height measurements were done using a graduated flexible nonextensible ribbon tape (Gulick Measuring Tape©) provided with a spring enabling the adjustment of the tension applied during measurement and having a precision of 0.1 cm. These measurements were done in accordance with the WHO recommendations [12].

#### 3.5.5. Body Mass Index (BMI)

Body mass indices were calculated using formula BMI = weight (kg)/height (m^2^). WHO thresholds were used to define general obesity, with BMI ≥ 30. Overweight was considered as BMI between 25 and 29 and underweight BMI < 18.5. BMI values between 18.5 and 24.9 were considered normal [13].

#### 3.5.6. Waist Circumference Measurement

Waist circumference as an indicator of abdominal obesity was measured in accordance with the WHO STEPS method using a graduated flexible nonextensible ribbon tape (*see height measurement*)

### 3.6. Ethical Considerations

The authorisation to carry out this survey was obtained from the Adamawa Regional Delegation of Public Health (ref. N°507/L/HN/DRSPA/SAGE/BPF/NGD) and the Ethical Committee of the Ngaoundéré Regional Hospital (ref. N°1233/L/RC/RA/DSP/HR/NGD/CLE). Participants provided written consent after obtaining information on the nature of the study and its merits and demerits. Specimen collection was performed under aseptic conditions and participant information was handled confidentially. Results per quarter were kept in the corresponding health centres, to be handed to the individual participants by the chief of the centre while ensuring proper referral for those with abnormal values.

### 3.7. Data Management, Analysis, and Interpretation of Results

All the data collected on the individual record sheet of participants were transferred onto an Excel spreadsheet. The confidentiality of the data was respected by minimising access to the data using a password. The saved data were verified with participants' individual record sheets for conformity and consistency. The data were finally forwarded onto SAS software version 9.1 for analysis with statistical significance stated at *p* < 0.05.

## 4. Results

### 4.1. Basic Characteristics of the Study Population

Our survey population was made of 700 adults, with an age range of 18 to 93 years and average age of 36 ± 17 years. Among them were 340 (48.6%) men and 360 (51.4%) women. Two-thirds of this population were made up of Muslims and a third of Christians. The majority of our population were housewives (32%) and were predominantly possessing secondary level education (41.1%). With respect to participants with more than secondary education, women were less educated than men (9.1% against 16.9%).

### 4.2. Distribution of Cardiovascular Disease (CVD) Risk Factors

#### 4.2.1. High Blood Pressure

A total of 143 (20.43%) of the study participants had BP values above the normal, comprising 9.57% men and 10.86% women. The prevalence of higher-than-normal BP was recorded as 19.71% and 21.1% among male and female participants, respectively. In individuals under the age of 30 years, 9.40% presented higher-than-normal blood pressure and likewise 9.76%, 27.59%, and 45.03% in the age groups 30–39 years, 40–49 years, and >50 years, respectively. Although wide differences were observed between subgroup population weights, divorced persons (53.33%) had the highest frequency of high blood pressure followed by married participants (25.0%). Subpopulation proportions of participants with high BP were the highest among the unemployed (34.29%), followed by household workers (29.91%), and were the least among civil servants (6.19%). Furthermore, frequency distributions of high blood pressure following educational level were performed and it was observed that 87.02% of persons with higher-than-normal blood pressure possessed at most a secondary level of education. The distributions of high blood pressure following sociodemographic characteristics are presented in Table 1.

#### 4.2.2. Obesity and Overweight

**Figure 2:**
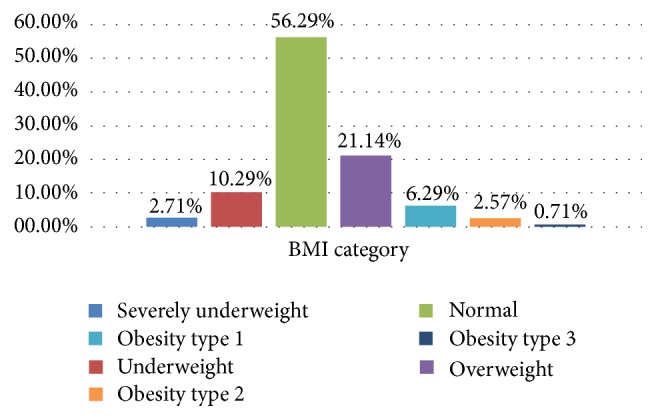
Distribution of study participants by BMI categories.

Overall, 21.14% of the participants were overweight and 9.57% of the individuals were obese (Figure 2). The distribution of obesity according to gender showed a prevalence of 3.29% among men and 6.28% among women.

#### 4.2.3. Other CVD Risk Factors

**Figure 3:**
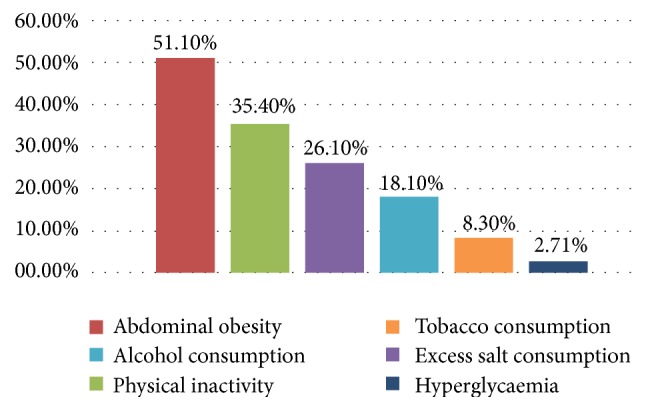
Distribution of other CVD risk factors in the study population.

The other CVD risk factors we studied include hyperglycaemia, abdominal obesity, physical inactivity, and excess salt, alcohol, and tobacco consumption. Among these, physical inactivity (35.4%) was highly prevalent, second only to abdominal obesity (51.1%). Hyperglycaemia was the least present in our study population (Figure 3).

**Figure 4:**
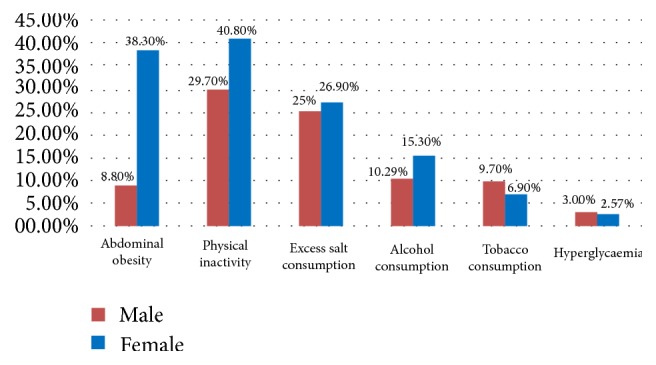
Distribution of other CVD risk factors by gender.

Gender based distributions demonstrated high frequencies of abdominal obesity, physical inactivity, and excess salt and alcohol consumption in women relative to men, while higher proportions of the male population were smokers and hyperglycaemic subjects (Figure 4).

#### 4.2.4. Notion of Family History and Hereditary Diabetes, Arterial Hypertension, and Obesity

The possibility for hereditary hypertension, diabetes, and obesity was defined and constituted persons with raised BP (paired), FBG, and BMI measurements, having family history (at least a parent or sibling diagnosed) of hypertension, diabetes, and obesity, respectively. The probability of hereditary (familial) hypertension, diabetes (using hyperglycaemia), and obesity was thus estimated at 11.6%, 11.28%, and 4.6%, respectively, in our survey.

### 4.3. Factors Associated with Hypertension

Based on blood pressure measurements and following JNC-7 recommendations [14], normotensive, prehypertensive, and hypertensive persons were estimated at 67.43%, 12.14%, and 20.43%.

Examining the distribution of high BP between other categories of CVD risk factors, hyperglycaemic persons (46.15%) exhibited the highest prevalence of high BP followed by obese participants (31.34%) and then tobacco consumers (25.86%) as shown in Table 2.

Regression analysis proved that advanced age, alcohol consumption, hyperglycaemia, and a divorced marital status were associated with hypertension. Participants aged 40 to 49 (OR = 3.83; *p* = 0.0016) and 50 years and above (OR = 7.08; *p* < 0.0001) were more likely to have high BP values compared to those under 30 years of age. Likewise, participants with hyperglycaemia compared to normoglycaemia (OR = 4.2; *p* = 0.027) and those divorced compared to unmarried ones (OR = 4.11; *p* = 0.0488) were more prone to high blood pressure values, while alcohol consumers compared to nonconsumers were less likely to have high BP levels (OR = 0.16; *p* = 0.0025) (Table 3).

## 5. Discussion

Except for some hospital studies involving a reduced number of participants and usually focussed on only one risk factor, few epidemiological data are available quantifying cardiovascular disease risk factors in northern regions of Cameroon. This community survey carried out in Ngaoundéré town was aimed at studying cardiovascular disease risk factors and their association with hypertension so as to contribute to the improvement of disease prevention, control, and management.

### 5.1. The Prevalence of Cardiovascular Disease Risk Factors

#### 5.1.1. Arterial Hypertension

Our study showed that approximately one-fifth (20.43%) of the study participants had arterial blood pressure values higher than normal at the time of measurement. Data from a 2004 study by Kengne et al. reported similar rates (20.8%) in Cameroon [15]. Studying a ten-year variation in hypertension in Cameroon, Fezeu et al. (2010) suggested a 2- to 5-fold increase in both urban and rural dwellers of both sexes [16]. The distribution of hypertension in sub-Saharan Africa varies considerably between countries with recent surveys reporting occurrence rates of 27.9% in Ethiopia [17], 31.8% in Nigeria [18], and 47% in Kenya [19], among others. As previously proposed by other authors, we acknowledge the contribution of socioeconomic, environmental, and behavioural determinants to this prevalence, including increasing economic growth and urbanisation, consumption of tobacco and fatty foods, and long periods of risk exposure to these factors due to a long-life experience [20, 21].

#### 5.1.2. Overweight and Obesity

Overweight and obesity were estimated in 21.14% and 9.57% of the study population, respectively, values which are lower than the 23.7% and 11.1% previously reported in Cameroon [15]. Although our results recorded the prevalence of overweight and obesity lower than the 34.5% and 9.5% observed in Kenya [22], 28.2% and 13.8% in Nigeria [18], and 29.6% and 13.0% in Iran [23], respectively, they remain far below the 72.4% recorded in South Africa in 2013 [24]. Gender based distribution of obesity portrayed women (6.28%) to be more affected by obesity than men (3.29%), concurring with previous findings [17, 19, 23]. Our results differ greatly and are much lower than those reported in Western Europe (France: 6.28% in men and 37% in women) and Asia (China: 45% in men and 32% in women) [25]. These differences could be explained by the improved living standards, advanced state of urbanisation, and other inherent sociocultural attributes associated with the latter region.

In addition, we observed that abdominal obesity occurred in about half of our participants (51.1%) in the study. Kengne and coworkers reported 14% and 59.5% prevalence of abdominal obesity in urban dwelling Cameroonian men and women, respectively [15]. Our rate, despite being in line with the reports of Elasmi et al. (2009) in the Grand Tunis population [26], is far higher than the 14.4% observed in Iran [23] and is possibly the result of an increase in the consumption of salts and fatty and energy-rich foods with deprivation in vitamins and mineral salts due to the change in lifestyle habits [27].

#### 5.1.3. Hyperglycaemia

Hyperglycaemia was defined as an increase in blood glucose level above 1.26 g/dL measured after an overnight fast of 8 to 12 hours with or without any associated clinical manifestations. The prevalence of hyperglycaemia was 5.57% in our survey (3% in men and 2.57% in women). Diabetes mellitus has been reported to occur in about 2.9% to 6.2% of Cameroon's rural and urban subpopulations [28], similar to the observed 3.6% in neighbouring Nigeria [18]. This is lower than the results reported in several countries: 11.9% in men and 11.7% in women in South Africa [24], 7.8% in men and 8.5% in women in Congo [29], 7.2% in men and 4.3% in women in France, and 12.6% in men and 9.1% in women in the United States [30]. Probably, our sampling methods could have led to underestimation of the occurrence of hyperglycaemia in our study population as we did not consider diabetic patients on treatment and having normal FBG levels as being hyperglycaemic contrary to the other authors [26, 29].

### 5.2. Other Cardiovascular Disease Risk Factors

#### 5.2.1. Tobacco and Alcohol

The prevalence of alcohol and tobacco consumption in our survey was 18.1% and 8.3%, respectively, with even much lesser rates reported in rural Kenya [19], findings which are lower than the previously reported 85% and 16% in urban Cameroon [16] and 55.8% and 13% in Nigeria [18]. The prevalence of these two risk factors varies between countries with an unequal distribution between men and women [17, 22]. Inherent sociocultural and economic factors possibly affect these habits in our setting such as the state of reduced urbanisation and the widely practiced Muslim faith.

#### 5.2.2. Excessive Salt Consumption

26.1% of participants consumed large quantities of salt, which is encouraged by several sociocultural practices. The high consumption of sodium is common in Kenya due to the use of salts to preserve food or render it appetising or to be added to prepared food by consumers [19]. These facts were reflected in our work as several participants from different quarters of the town were observed to consume a large quantity of salts.

#### 5.2.3. Sedentary Lifestyle

Compared to the 35.4% proportion of our participants characterised with a sedentary lifestyle, other African studies have reported relatively higher values of 52.2% [24] and 42.6% [29]. This is likely owing to the fact that animal breeding and farming which are most practiced in this region necessitate important geographical displacement. In our study, however, physical inactivity appeared to be a more dominant risk factor for cardiovascular diseases compared to high blood pressure, thus contradicting reports of other sub-Saharan studies in which arterial hypertension was observed to be the most prevalent CVD risk factor [22, 30].

#### 5.2.4. Hereditary Hypertension, Diabetes, and Obesity

The probability of family heredity of arterial hypertension was found in 11.6% of the study population and likewise 11.28% and 4.6% for diabetes and obesity, respectively. It has been clearly established in previous studies that family history of arterial hypertension is associated with a significant risk of hypertension for both men and women [31]. Our results therefore suggest a relatively high medium- to long-term risk of hypertension in our population.

### 5.3. Factors Associated with Arterial Hypertension

Previous reports have established the role of advanced age as a risk factor for hypertension [17, 24, 32]. A study carried out in Israel between 2002 and 2007 showed that ten years caused a 1.6-fold rise in the risk of being in a higher JNC-7 category of blood pressure [33]. Regression analysis reiterated the relationship between advanced age and arterial hypertension, as the likelihood of high blood pressure was 3.8-fold (*p* = 0.0016) and 7.1-fold (*p* < 0.0001) the normal for participants aged 40–49 and 50 years and above, respectively, compared to those 30 years of age and below.

Hyperglycaemia is another CVD risk factor which demonstrated an association with hypertension in this study. 46.15% of the participants with raised fasting blood sugar levels had higher-than-normal blood pressure values. These findings are in conformity with the previous literature [34].

Alcohol consumption was inversely associated with arterial blood pressure: consumers of alcohol were less likely to have high BP values compared to nonconsumers (*p* = 0.0025; OR = 0.16). These results are similar to those obtained by Peltzer and Phaswana-Mafuya [24], although other studies hold the notion that the consumption of even very little quantities of alcohol increases the risk of hypertension [35, 36].

### 5.4. Limitations of This Study

This study was characterised with several limitations with the most significant being the reliability of data analysis based on nonverifiable verbal responses of participants. Also, the evaluation of alcohol and tobacco consumption was incoherent as several participants consumed tobacco in powder form by chewing and others consumed local alcoholic beverages rendering quantification difficult. Lastly, the time frame within which our study was carried out did not permit an elaborate investigation on dietary awareness, patterns, and practice as a function of CVD risk at individual level within the population.

### 5.5. Conclusion

Our study on cardiovascular diseases risk factors indicates relatively high occurrences of several factors within the Ngaoundéré community with dominant risk factors being abdominal obesity, physical inactivity, excessive salt consumption, arterial hypertension, and alcohol consumption. Furthermore, advanced age, hyperglycaemia, a divorced marital status, and consumption of alcohol were associated with higher-than-normal blood pressure levels.

### 5.6. Perspectives

This study was carried out in an urban setting within the Adamawa Region where access to health information, services, and promotion programs and socioeconomic and educational levels are relatively higher compared to rural settlements. It is imperative therefore for future studies to be carried in rural communities of the Adamawa Region as well as comparative studies between regions of the Cameroon national territory. This will furnish relevant data necessary for informed decision-making, advocacy, elaboration of health promotion programs, and strengthening the health system in a bid to progressively and sufficiently prevent, control, and manage cardiovascular diseases nationwide.

## Figures and Tables

**Table 1:** Distribution of high blood pressure by age, marital status, and gender.

Variable	Total, *n*	High blood pressure			
		Men, *n* (%)	Women, *n* (%)	Total, *n* (%)	Proportion of the study population (%)
Age (years)					
<30	319	18 (5.64)	12 (3.76)	30 (9.40)	4.29
30–39	123	2 (1.63)	10 (8.13)	12 (9.76)	1.71
40–49	87	13 (14.94)	11 (12.64)	24 (27.59)	3.43
≥50	171	34 (19.88)	43 (25.15)	77 (45.03)	11.00
Marital status					
Unmarried	253	16 (6.32)	10 (3.95)	26 (10.28)	3.71
Married	426	46 (10.80)	59 (13.85)	105 (24.65)	15.00
Widowed	6	3 (50.00)	1 (16.67)	4 (66.67)	0.57
Divorced	15	2 (13.33)	6 (40.00)	8 (53.33)	1.14
Profession					
Farmers	34	2 (5.88)	2 (5.88)	4 (11.76)	0.57
Drivers	26	5 (19.23)	0 (0.00)	5 (19.23)	0.71
Traders	139	19 (13.67)	3 (2.16)	22 (15.83)	0.03
Students	125	9 (7.20)	7 (5.60)	16 (12.80)	2.28
Household workers	224	22 (9.82)	45 (20.09)	67 (29.91)	9.57
Civil servants	117	0 (0.00)	7 (5.98)	7 (6.19)	1.00
Unemployed	35	10 (28.57)	2 (5.71)	12 (34.29)	1.71
Level of education					
Uneducated	92	3 (3.26)	8 (8.70)	11 (11.96)	1.57
Coranic	107	16 (14.95)	18 (16.82)	34 (31.78)	4.86
Primary	121	8 (6.61)	20 (16.53)	28 (23.14)	4.00
Secondary	288	32 (11.11)	26 (9.03)	58 (20.14)	8.29
Higher	92	8 (8.70)	4 (4.35)	12 (13.04)	1.71
Religion					
Atheist	2	0 (0.00)	0 (0.00)	0 (0.00)	0.00
Christian	235	17 (7.23)	18 (7.66)	35 (14.89)	5.00
Muslim	463	50 (10.80)	58 (12.53)	108 (23.33)	15.42

**Table 2:** Distribution of High BP between other CVD risk factors and gender.

CVD risk factors	Total, *n*	High blood pressure			
		Men, *n* (%)	Women, *n* (%)	Total, *n* (%)	Proportion of study population (%)
Hyperglycaemia	39	9 (23.08)	9 (23.08)	18 (46.15)	2.57
Obesity	67	15 (22.39)	6 (8.96)	21 (31.34)	3.00
Alcohol consumption	127	8 (6.30)	5 (3.94)	13 (10.24)	1.86
Tobacco consumption	58	7 (12.07)	8 (13.79)	15 (25.86)	2.14
Physical inactivity	248	21 (8.47)	38 (15.32)	59 (23.79)	8.43
Excessive salt consumption	182	12 (6.59)	24 (13.19)	36 (19.78)	5.14

**Table 3:** Logistic regression for HBP with other CVD risk factors.

Variables	*p* value	Odds ratio	[95% CI]
Age group			
<30	Reference	1	
30–39	0.8419	0.91	[0.36–2.2]
40–49	0.0016^*∗∗*^	3.83	[1.66–8.9]
≥50	<0.0001^*∗∗∗*^	7.08	[3.43–15.2]

Sex			
Male	Reference	1	
Female	0.6820	0.77	[0.35–1.68]

Obesity			
No	Reference	1	
Yes	0.0881	1.04	[0.049–0.22]

Alcohol consumption			
No	Reference	1	
Yes	0.0025^*∗∗*^	0.16	[0.046–0.49]

Tobacco consumption			
No	Reference	1	
Yes	0.0561	2.26	[0.95–5.18]

Physical inactivity			
No	Reference	1	
Yes	0.3680	1.03	[0.28–3.70]

Excessive salt consumption			
No	Reference	1	
Yes	0.1450	1.05	[0.52–2.07]

Hyperglycaemia			
No	Reference	1	
Yes	0.0270^*∗*^	4.20	[1.17–15.2]

Marital status			
Unmarried	Reference	1	
Divorced	0.0488^*∗*^	4.11	[0.92–18.3]
Married	0.8634	0.80	[0.39–1.62]
Widowed	0.7964	1.2	[0.13–12.5]

^*∗∗∗*^ *p* < 0.001; ^*∗∗*^ *p* < 0.01; ^*∗*^ *p* < 0.05.
